# The Effect of Ginkgo Biloba Dropping Pills on Hemorheology and Blood Lipid: A Systematic Review of Randomized Trials

**DOI:** 10.1155/2019/2609625

**Published:** 2019-06-26

**Authors:** Hong Chen, Cihang Zhou, Mingwei Yu, Shuo Feng, Yunfei Ma, Zhengrong Liu, Jiahui Zhang, Tongjing Ding, Bo Li, Xiaomin Wang

**Affiliations:** ^1^Beijing Hospital of Traditional Chinese Medicine, Capital Medical University, Beijing 100010, China; ^2^Beijing University of Traditional Chinese Medicine, Beijing 100029, China; ^3^Changhai Hospital Affiliated to Naval Military Medical University, Shanghai 200433, China; ^4^Beijing Institute of Traditional Chinese Medicine, Beijing 100010, China; ^5^Yanqing Hospital of Beijing Hospital of Traditional Chinese Medicine, Capital Medical University, 102100, China

## Abstract

**Objective:**

A systematic review of randomized trials was performed to assess the effect of Ginkgo Biloba Dropping Pills (GBDP) on clinical hemorheology and blood lipid indicators.

**Methods:**

The data of the Embase, Cochrane Library, PubMed, Clinical Trials, China National Knowledge Infrastructure, the Wanfang database, the VIP database, and the Sinomed were retrieved by computers from the establishment of the database to March 27, 2018, and screened and extracted by two researchers according to inclusion and exclusion criteria. Cochrane 5.0 recommended bias risk assessment tool was used to evaluate the methodological quality of the included literature, and Revman 5.3 software were used for meta-analysis.

**Results:**

10 literatures were finally selected in accordance with the standard. There were a total of 1201 cases, 608 cases in ginkgo biloba dropping pill group and 593 in routine treatment group. Compared with control group, GBDP significantly improved plasma viscosity [N=383, RR= - 0.45, 95%CI=(-0.86,-0.04), P=0.03], whole blood high shear [N=232, RR= - 0.92,95%CI=(-1.69, -0.16), P =0.02], whole blood low shear [N = 232, RR = - 2.22, 95% CI = (- 3.74, -0.7), P = 0.004], red blood cell specific volume [N =132, RR = - 4.55, 95% CI = (- 6.36, 2.73), P < 0.000 01], fibrinogen [N=243, RR=-0.60,95%CI=(-0.82,-0.39), P<0.00001], triglyceride [N=912, RR=-0.60,95%CI=(-1.12, -0.07), P =0.03], cholesterol [N=912, RR=-0.97,95%CI=(-1.41, -0.52), P <0.0001], low-density cholesterol [N=1100, RR=-0.72,95%CI=(-1.19, -0.25), P =0.003], and sensitivity analysis before and after of high-density cholesterol [N=1020, RR=0.08,95%CI=(-0.17,0.34), P =0.52] and [N=683, RR=0.27,95%CI=(0.13,0.42), P =0.0003]. And seven adverse reactions were reported.

**Conclusion:**

GBDP can improve hemorheology indexes, which is to reduce the blood viscosity, to improve blood lipid status, and to prevent and treat cardiocerebral and renal vascular diseases to a certain extent, with slight clinical adverse reactions. But our results were based on small amount of clinical studies with poor quality and insufficient evidence, which may lead to low credibility of conclusions. Therefore, more large-sample, multiple-center, randomized controlled clinical trials and related mechanisms researches are needed to obtain better clinical trial evidence in order to verify the further effectiveness and safety of GBDP on hemorheology.

## 1. Introduction 

Hemorheology is the flow properties of blood. The core role lies in the change of its index before the occurrence of vascular disease [1]. It is a crucial clue for the etiology, diagnosis, prevention, treatment, therapeutic observation, and disease monitoring of many diseases and an important tool that is indispensable in clinical medicine and scientific research [2, 3]. Hemorheology indicators mainly include whole blood viscosity, plasma viscosity, hematocrit, and so on [4], which are commonly used for the detection of vascular diseases such as coronary heart disease, hypertension, and stroke [5, 6]. Current studies have shown that blood viscosity can be used as an alarm signal for coronary heart disease and myocardial infarction [7], and the change of whole blood viscosity is closely related to the total mortality of end-stage kidney [8]. In addition, hemorheology is an important marker of prognosis of stroke patients [9], which is possible to become an important indicator of tumor occurrence and metastasis [10–12] and play a positive role in the clinical diagnosis of first-episode schizophrenia [13]. Some studies have shown that blood lipid testing can be used as the basis for the diagnosis of diabetes [14], which can effectively improve the clinical diagnosis rate and judge the progress of cardiovascular and cerebrovascular diseases [15]. And abnormal lipid levels are associated with the occurrence and development of colorectal cancer and breast cancer [16, 17]. It has been suggested in the literature that the blood lipids and hemorheology tests have significant effects in the diagnosis of cardiovascular diseases [18], and they play a very important role in the diagnosis, treatment, and efficacy observation of cerebrovascular diseases [19]. The degree of pathological changes is related to the stage, prognosis of the disease, and the risk of thrombosis in patients [20–23].

Studies have shown that ginkgo biloba extract can improve hemorheology [24], reduce blood lipid [25], inhibit platelet aggregation [9], efficiently cross the blood-brain barrier [24], and so on. GBDP is a new type of preparation of ginkgo biloba extract (full extract) on the extraction method of the preparation of production according to the Pharmacopoeia of the People's Republic of China (Edition 2015). It not only keeps the active ingredients, but also increases the concentration of the active ingredients, which has the characteristics of rapid clinical effect, good efficacy, small dose, taking safety, good patient compliance, and long-term administration [26]. Studies have shown that blood stasis is positively correlated with hemorheology [27]. According to the Chinese Pharmacopoeia, GBDP have the function of activating blood circulation, removing blood stasis, and activating channels and collaterals [28], which can be widely used in various diseases of blood stasis and blocking collaterals. At present, there are some problems in the study of hemorheology and lipid in GBDP, such as small sample size, unclear efficacy, and inconsistent conclusions. Therefore, meta-analysis was used to analyze the results of relevant clinical trials and evaluate the effects of GBDP on hemorheology and blood lipid, so as to provide more reliable evidence-based medical evidence for clinical practice.

## 2. Methods

### 2.1. Search Strategy

The computer retrieved the data from China National Knowledge Internet, Wanfang Database, Weipu Network, China Biomedical Literature Database, Cochrane, PubMed, and Clinical Trials databases, and the search was made from the establishment of the database to March 27, 2018. The Chinese search term was “Ginkgo Biloba Dropping Pills”, and the English search terms included “ginkgo”, “ginkgoes”, “yinxingye”, “pill”, and “pills”. The keywords were combined with free words and Boolean logic words (AND, OR, and NOT) were used. The combined method was adjusted according to different databases, all the search strategies were preexperimented several times, and the one with the highest number of documents was finally selected.

### 2.2. Inclusion and Exclusion Criteria

#### 2.2.1. Inclusion Criteria

The inclusion criteria are as follows: (1) Subjects: randomized controlled trials with unlimited disease types, limited to Chinese and English. (2) Interventions: the control group was treated with placebo or conventional therapy; the treatment group was treated with GBDP or conventional treatment combined with GBDP. (3) Outcome indicators: ① whole blood low cut viscosity, ② whole blood high cut viscosity, ③ plasma viscosity, ④ hematocrit, ⑤ fibrinogen, ⑥ cholesterol, ⑦ low-density lipoprotein, ⑧ high-density lipoprotein, ⑨ triglyceride, and ⑩ adverse reactions; the main outcome indicators include at least one from ①~⑤.

#### 2.2.2. Exclusion Criteria

The exclusion criteria are as follows: (1) The test group consists of 3 groups or more. (2) The treatment group adds 2 or more interventions based on the control group. (3) Review, cohort study, case-control, case report, retrospective study, etc. (4) Duplicate documents. (5) Data missing and inaccessible.

### 2.3. Literature Screening and Data Extraction

The search strategy was developed and searched by two experienced evaluators. According to the preset inclusion and exclusion criteria, the topics and abstracts were read according to a unified process, and the articles were excluded that do not meet the inclusion criteria. Record the first excluded literature and reasons; fatherly obtain the full text of the documents that meet the inclusion criteria, read the full texts to judge whether it met the inclusion criteria, record the second excluded literature and reasons, and finally cross-check the results; the disputed documents were resolved through discussion between the two parties, and they were decided by a third party if not resolved. Extract the data of the final included documents, set the data extraction form, and fill in the data extraction form. The form included the following contents: (1) the patient's age, gender, duration of the disease, and other baseline data; (2) the author's name; year of publication; (3) Research background, including research provinces, hospitals, and fund support; (4) research design schemes, comparison methods and types of research, sample size calculation, randomization methods, interventions, intervention courses, number of participants, number of people lost, etc.; (5) outcome indicators.

### 2.4. Quality Evaluation

Using the migration risk assessment tool recommended by the Cochrane Handbook 5.1.0, methodological quality evaluation of the included literature includes the following: (1) random assignment method; (2) covert grouping: (3) blind method; (4) the integrity of the resulting data; (5) selective reporting: (6) other sources of bias. Each aspect was evaluated by “low risk”, “unclear”, and “high risk” and was independently completed by two evaluators and then cross-checked. If the opinions were not unified, the two sides would be discussed and decided. If still undecidable, there would be a ruling by a third party with higher qualifications.

### 2.5. Statistical Processing

Statistical analysis was performed by the dedicated software Revman 5.3 provided by the Cochrane Collaboration Net. First, the clinical heterogeneity was assessed. If there was no clinical heterogeneity, the chi-square test was used to test the heterogeneity of the results of the relevant literature. If P ≧ 0.10, I^2^ ≦ 50%, there was no statistical heterogeneity between the results included in the study; the fixed effect model was used for analysis: if p < 0. 10, I^2^ > 50%, it was considered as statistically different. Then we made an analysis on the heterogeneity sources to determine whether the sensitivity analysis and subgroup analysis were needed, whether random effects models can be used for analysis or not. Descriptive analysis was used if the clinical heterogeneity was too large or the literature was too few to perform meta-analysis. If the result was a binary categorical variable, the relative risk (RR) was used as the analytical statistic, if it was a continuous variable; the mean and standard deviation (WMD) were used as the analytical statistics. The interval estimate was expressed as 95% confidence interval (CI), and a forest map was drawn. Methodological quality evaluation was performed by the bias risk map drawn by Revman 5.3 software. For more than 10 articles on a certain outcome indicator, an inverted funnel plot was used to assess the published bias of the included study.

## 3. Results

### 3.1. Literature Search Results

**Figure 1:**
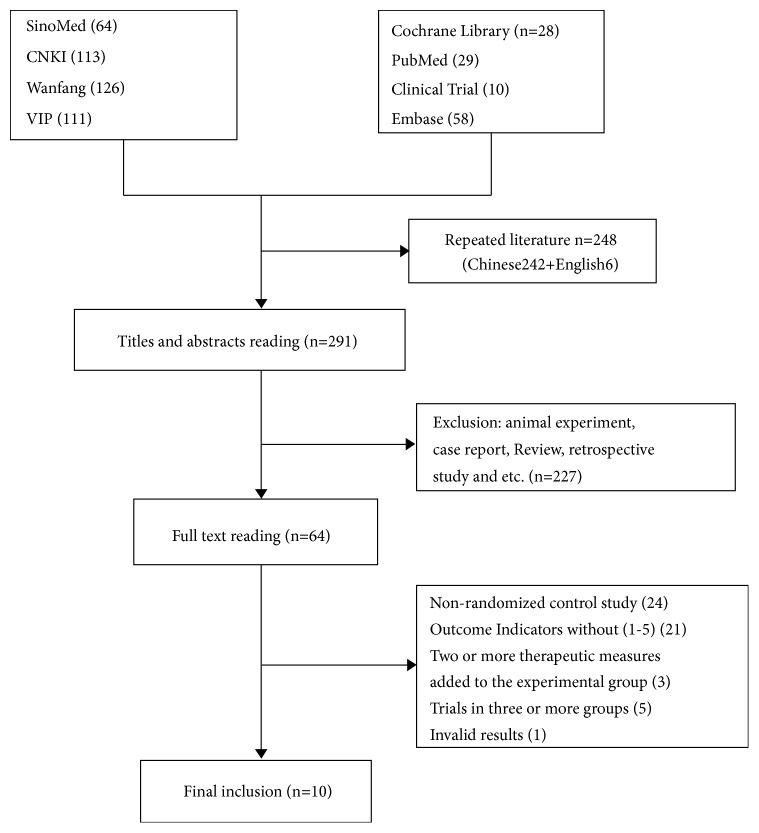
Document screening process.

A total of 539 literature titles were retrieved by computer and manual retrieval methods. The search was carried out according to the corresponding search formulas. A total of 125 English documents were retrieved, and none was eventually included. A total of 414 Chinese literatures were searched and 10 articles in the full text were finally included for meta-analysis after gradually screened. The screening process in the study was shown in Figure 1.

### 3.2. Basic Information for Inclusion in Research

Finally, 10 randomized controlled trials were included; a total of 1201 cases were reported. There were 608 cases in GBDP group and 593 in control group. The publication period was from 2008 to 2017. Five studies included the main outcome indicators, and one did not indicate the treatment cycle. Basic information for inclusion in the study was shown in Table 1.

### 3.3. The Results of Methodological Quality Assessment Are Shown in Figures 2 and 3

One of the ten studies was randomized for the order of visits. One study was randomized and two studies were nonequally randomized. Others did not show specific methods. None of the studies described the allocation of concealment methods, and none of the placebo controls were used. Only one report was a single-blind study, seven articles reported adverse reactions, and only one reported shedding and rejection.

### 3.4. Evaluation of Efficacy

#### 3.4.1. Plasma Viscosity

**Figure 4:**
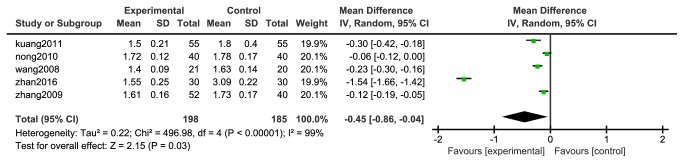
Meta-analysis forest map of plasma viscosity.

Five [29–34] studies reported changes in plasma viscosity, 248 cases in the experimental group and 235 in the control group, with high homogeneity between the studies (P <0.00001, I^2^= 99%), so random effects model was used. The analysis was detailed in Figure 4. The results of meta-analysis showed that the difference between the experimental group and the control group was statistically significant [N=383, RR=-0.45, 95%CI=(-0.86, -0.04), P =0.03].

#### 3.4.2. The Value of High Cut Blood Viscosity

**Figure 5:**
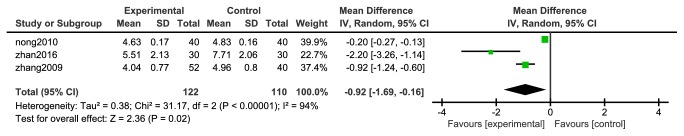
Meta-analysis forest map of the value of high cut blood viscosity.

Three [29–31] studies reported changes in the value of high cut blood viscosity, 172 cases in the experimental group and 160 in the control group; the heterogeneity between the studies was large (P <0.00001, I^2^= 94%), so the random effect model was adopted, as shown in Figure 5. The meta-analysis showed that the difference between the patients in the experimental group and the control group was statistically significant [N=232, RR=-0.92., 95%CI=(-1.69, -0.16), P =0.02].

#### 3.4.3. The Value of Low Cut Blood Viscosity

**Figure 6:**
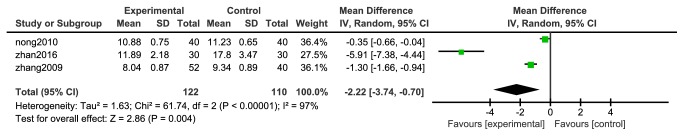
Meta-analysis forest map of the value of low cut blood viscosity.

Three [29–31] studies reported changes in the value of low cut blood viscosity, including 122 cases in the experimental group and 110 in the control group. There was a high degree of homogeneity between the two studies (P =0.49, I^2^= 0%), so the random effect model was adopted, as shown in Figure 6. The meta-analysis showed that the difference between the experimental group and the control group was statistically significant [N=232, RR=-2.22, 95%CI=(-3.74, -0.7), P =0.004].

#### 3.4.4. Hematocrit

**Figure 7:**
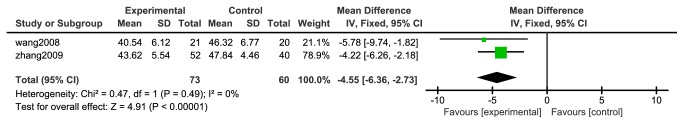
Meta-analysis forest map of hematocrit.

Two [31, 32] studies reported changes in hematocrit, 73 cases in the experimental group and 60 in the control group. The two studies were highly homogenous (P =0.49, I^2^= 0%), so that a fixed effect model was used, as shown in Figure 7. The results of meta-analysis showed that the difference between the experimental group and the control group was statistically significant [N=133, RR=-4.55, 95%CI=(-6.36, -2.73), P <0.00001].

#### 3.4.5. Fibrinogen

**Figure 8:**
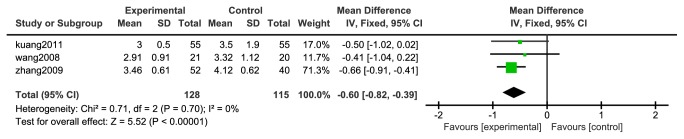
Meta-analysis forest map of fibrinogen.

Three articles [31–33] reported changes in fibrinogen, 178 cases in the experimental group and control group. In two studies, there was good homogeneity between the two studies (P <0.0001, I^2^= 0%), so a fixed effect model was used, as shown in Figure 8. The meta-analysis showed that the difference between the experimental group and the control group was statistically significant [N=243, RR=-0.60, 95%CI=(-0.82, -0.39), P <0.00001].

#### 3.4.6. Triglyceride (TG)

**Figure 9:**
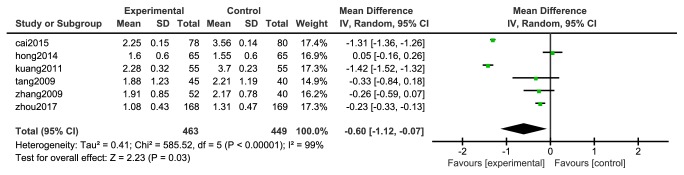
Meta-analysis forest map of triglyceride.

Six [31, 33–37] studies reported changes in triglycerides, 463 cases in the experimental group and 449 in the control group; the heterogeneity between the two studies was large (P <0.00001, I^2^= 99%), so a random effects model was used for analysis, as shown in Figure 9. The results of meta-analysis showed that the difference between the experimental group and the control group was statistically significant [N=912, RR=-0.60, 95%CI= (-1.12, -0.07), P =0.03].

#### 3.4.7. Cholesterol (TC)

**Figure 10:**
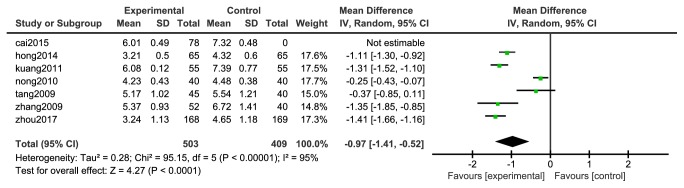
Meta-analysis forest map of cholesterol.

Seven [29, 31, 33–37] studies reported changes in cholesterol (TC), 503 cases in the experimental group and 409 in the control group. The heterogeneity between the two studies was large (P <0.0001, I^2^= 95%), so a random effect model was adopted, as shown in Figure 10. The meta-analysis showed that the difference between the experimental group and the control group was statistically significant [N=912, RR=-0.97, 95%CI= (-1.41, -0.52), P <0.0001].

#### 3.4.8. Low-Density Lipoprotein Cholesterol (LDL-C)

**Figure 11:**
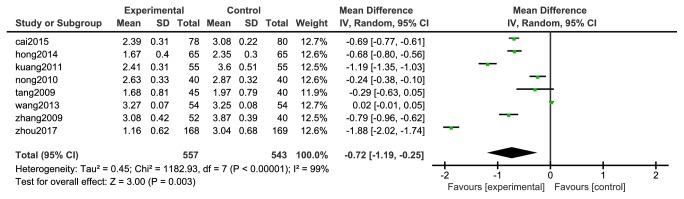
Meta-analysis forest map of LDL-C.

Eight [29, 31, 33–38] studies reported changes in low-density lipoprotein (LDL), 557 cases in the experimental group and 543 in the control group, the heterogeneity between the two studies was large (P <0.00001, I^2^= 99%), so a random effect model was used for analysis, as shown in Figure 11. The meta-analysis showed that the difference between the experimental group and the control group was statistically significant [N=1100, RR=-0.72, 95%CI= (-1.19, -0.25), P =0.003].

#### 3.4.9. High-Density Lipoprotein Cholesterol (HDL-C)

**Figure 12:**
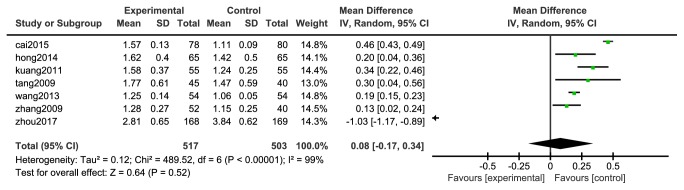
Meta-analysis forest map of HDL-C.

Seven [31, 33–38] studies reported changes in high-density lipoprotein (HDL), 517 cases in the experimental group and 503 in the control group. The heterogeneity between the two studies was large (P <0.00001, I^2^= 99%), so a random effect model was used for analysis, as shown in Figure 12. The meta-analysis statistics spanned across invalid lines; the difference between the two groups was not statistically significant [N=1020, RR=0.08, 95%CI= (-0.17, 0.34), P =0.52].

#### 3.4.10. Adverse Reactions

Seven [30–33, 36–38] studies reported adverse events, and two studies [30, 32] reported a total of 3 adverse events in the treatment group; two studies [30, 38] in the control group reported 14 patients' adverse reactions. Four studies [31, 33, 36, 37] reported that no significant adverse reactions were found in either group.

#### 3.4.11. Funnel Chart

Since there were no more than 10 literatures with one outcome indicator, the drawing of inverted funnel diagram had not been carried out.

## 4. Discussion

**Figure 13:**
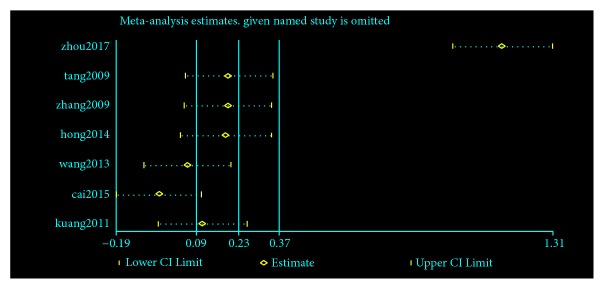
Sensitivity analysis of excluding individual studies.

**Figure 14:**
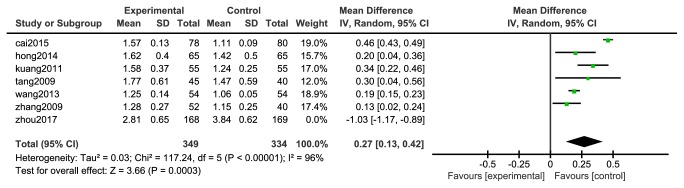
Meta-analysis forest map after eliminating HDL-C.

This study strictly followed the inclusion of exclusion criteria, followed the evidence-based medicine related research norms, and evaluated the meta-analysis of the effects of GBDP on hemorheology. Among the HDL-C results, only one study [34] was heterogeneous with other studies, so the sensitivity analysis was carried out. As shown in Figure 13, the first study had a greater impact on the total effect. If this study was excluded, the meta-analysis results were shown in Figure 14. The difference between the two groups was statistically significant [N=683, RR=0.27, 95%CI= (0.13, 0.42), P =0.0003].

The results showed that GBDP can obviously improve blood hemorheology to a certain extent, especially in hematocrit and fibrinogen; it can significantly reduce triglyceride, cholesterol and low-density lipoprotein cholesterol. It may increase the high-density lipoprotein cholesterol index, with mild adverse reactions. Therefore, GBDP can improve blood rheology and blood lipids to some extent and had certain protective effects on vascular function and no obvious adverse reactions were observed.

In many diseases, the increase of hemorheology and lipid index indicates that blood is in a thick and viscous state, which is prone to blood stasis. In view of “blood stasis”, the traditional Chinese medicine (TCM) adopts the method of activating blood and removing blood stasis. Current research shows that most of TCM for activating blood circulation have certain effect in the blood rheology, blood viscosity, platelet function, anticoagulation, protecting vascular endothelial cells, regulating blood lipid, etc., which has been widely applied in the treatment of cardiovascular diseases of its unique advantages in reducing the incidence of ischemic cerebrovascular disease, mortality and morbidity. What is more, compared with other drugs, the adverse reaction is small and relatively safe and has potential application prospect in the aspect of prevention, treatment, and rehabilitation [39]. Ginkgo leaf drop pill is a kind of traditional Chinese medicine to promote blood circulation and remove blood stasis, which has been widely used in neurology, cardiology, endocrinology, and respiratory medicine.

Ginkgo biloba extract has complex components; more than 160 kinds have been found so far, and its active components are mainly ginkgo flavonoids and ginkgo lactolides. According to the different extraction solvent, there are mainly three preparations of GBDP, ginkgo biloba extract tablets, and ginkgo ketone ester drop pills. The content of flavanols in GBDP is significantly higher than the other two preparations [40]. Research shows that flavonoids ingredients with expand coronary blood vessels improve blood vessels endings and relieve cerebral circulation, lower cholesterol, smooth muscle spasm, etc. [26]. It has been reported that GBDP can not only increase the blood flow of coronary arteries and cerebral blood flow, but also have a protective effect on myocardium and brain tissue under ischemia and hypoxia, reduce whole blood viscosity, plasma fibrinogen, TG, and ldl-c, and increase hdl-c, so as to improve hemorheology [41]. Although there is no direct or indirect evidence to indicate whether ginkgo biloba extract will lead to bleeding [24], it should be used sparingly or with caution if there is no obvious blood stasis to obstruct collateral due to the risk of causing bleeding.

Currently, there is no systematic review or meta-analysis of the effects of GBDP on hemorheology, only one systematic review on the effect of ginkgo biloba extract on hemorheology, which shows that ginkgo biloba extract can reduce blood and plasma viscosity and improve hemorheology in early diabetic nephropathy [42]. However, hemorheology is the second most important therapeutic index, and the changes of hematocrit, fibrinogen, and lipid are not reported. The study subjects were only diabetic nephropathy patients, and only two studies were included. Moreover, the extract of ginkgo biloba was not GBDP, which could not reflect the true efficacy of GBDP. Presently, there are potential pharmacodynamic differences among different types of ginkgo biloba preparations, but there is no specific study on the pharmacodynamic differences [40]. Therefore, the study of GBDP on hemorheology and lipid pharmacodynamics has a certain innovative significance.

However, there are some limitations in the inclusion of this study: (1) Insufficient search, only Chinese and English literature are restricted, yet other languages are not searched. (2) The quality of clinical trials included is low, and 7 studies do not describe the method of generating random sequences. The hiding of the whole studies' allocation program was not described, and sufficient information was not given to judge the quality of the studies. (3) No subgroup analysis of age and gender, the results lack certain representativeness. (4) The inconsistent treatment methods in the control group and the difference in drug dosage may have an impact on the evaluation of efficacy and safety. (5) The in-homogeneity of disease and treatment included in the study have not been able to evaluate the long-term efficacy. (6) Other hemorheology outcome indicators, such as erythrocyte aggregation coefficient, platelet adhesion, and platelet aggregation function, have not been included in hemorheology, so results need to be further improved.

## 5. Conclusion

In summary, in patients with hemorheology and dyslipidemia, GBDP can be taken individually or additionally, especially suitable for those with high hematocrit, fibrinogen, and cholesterol. To a certain extent, it plays a role in preventing and treating cardiocerebral and renal vascular diseases. However, our results are based on published studies, the number of included studies is small, and the quality is poor, which may lead to low credibility of conclusions. Currently blood rheology has not reached universal popularity in clinical applications, but there are good research prospects from this paper. Therefore, in the future, more large-scale, multicenter randomized controlled clinical trials and researches on related mechanisms should be implemented in a scientifically designed manner, more outcome indicators should be selected, longer treatment and follow-up observation should be conducted, and better clinical trial evidence should be obtained to fatherly verify the efficacy and safety of GBDP in hemorheology and vascular diseases.

## Figures and Tables

**Figure 2:**
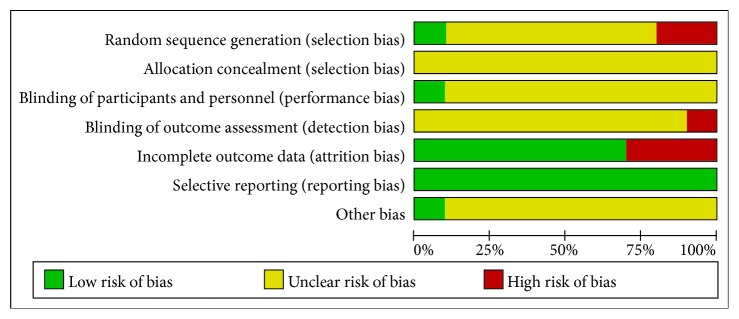


**Figure 3:**
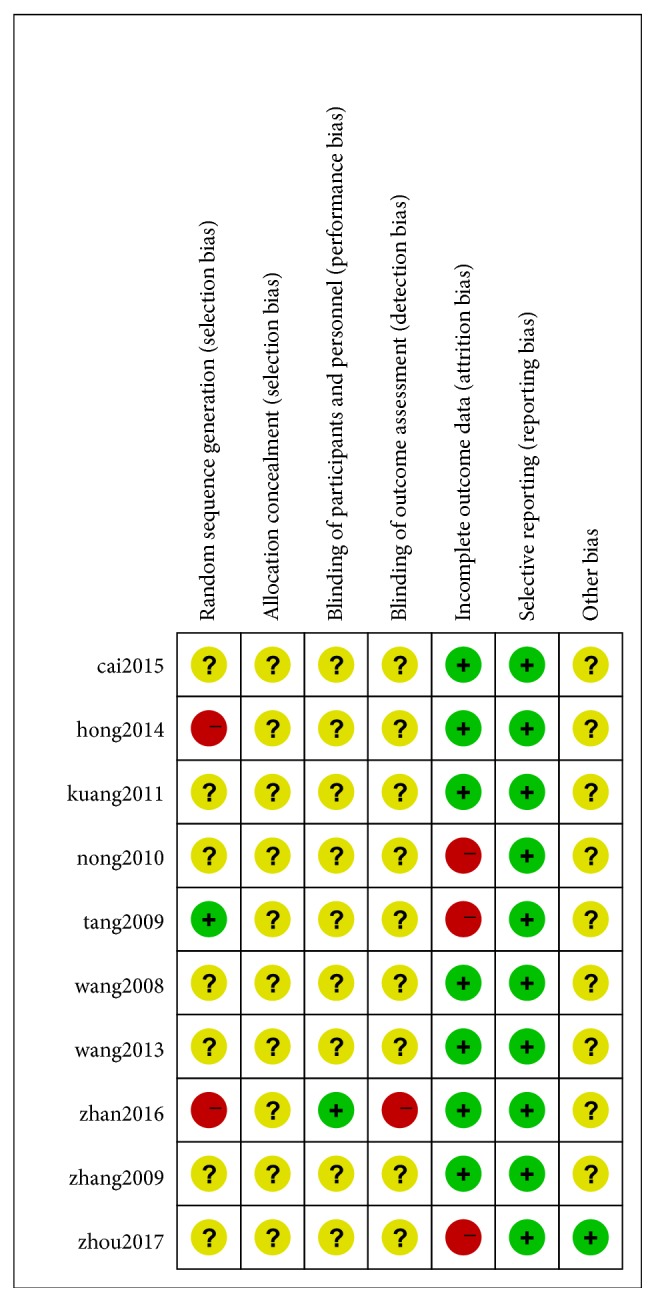


**Table 1:** Basic information for inclusion in the study.

First author, year	gender (male/female)		Mean age (years)		Intervention		Disease	course	Outcome indicator
	T	C	T	C	T	C			
Kuang weiwen 2011	ukn	ukn	ukn	ukn	GBDP, 300mg, po, tid. Nitroglycerin is prescribed during episode of angina.	Isosorbide nitrate, Aspirin enteric-coated tablets, Nitroglycerin is prescribed during episode of angina.	Unstable angina	4 w	③⑤⑥⑦ ⑧⑨ ⑩

Nong Hanglin 2010	25/15	30/10	66.55	65.38	GBDP, 315mg, po, tid. Take atorvastatin tablets	Take atorvastatin tablets	Acute cerebral infarction	4 w	①②③⑥⑦

Wang huigai 2008	11/10	11/9	61	60	GBDP, 180mg, po, tid. Routine dehydration, anticoagulation, antihypertensive, anti-infective, and supportive treatment	Routine dehydration, anticoagulation, antihypertensive, anti-infective, and supportive treatment	Cerebral infarction	1 m	③④⑤ ⑩

Zhang hong 2009	36/14	30/10	66.7	67 .1	GBDP, 180mg, po, tid. Aspirin, Cytidine diphosphocholine	Compound danshen pain, Aspirin, cytidine diphosphocholine	Multi-infarct dementia	4 w	①②③④ ⑤⑥⑦⑧⑨⑩

Zhan hong jing 2016	18/12	16/14	42.2	41.8	GBDP, 315mg, po, tid. Conventional treatments such as anti-infection, oxygen inhalation, phlegm and asthma relieving.	Conventional treatments such as anti-infection, oxygen inhalation, phlegm and asthma relieving.	Chronic obstructive pulmonary disease	14 d	① ②③ ⑩

Zhou jun 2017	86/94	72/108	72.43	74.67	GBDP, 315mg, po, tid. Oral hypoglycemic drugs or insulin injection	Oral hypoglycemic drugs or insulin injection	Elderly diabetes with atherosclerosis	12 w	⑥⑦⑧⑨

Tang zhihao 2009	ukn	ukn	53	57	GBDP, 315mg, po, tid. Fenofibrate, Pioglitazone hydrochloride	Fenofibrate, Pioglitazone hydrochloride	Nonalcoholic fatty liver	3 m	⑥⑦⑧⑨

Hong yan 2014	66/64	31/29	70.4	70.3	GBDP, 300mg, po, tid. Amlodipine besylate tablet	Amlodipine besylate tablet	Elderly hypertension	12 w	⑥⑦⑧⑨

Wang shengbao2013	32/22	26/28	50	53.5	GBDP, 315mg, po, tid.	Isosorbide nitrate, enteric-coated aspirin	Coronary heart disease	60 d	⑦⑧

Cai lingqin 2015	148/30	52/26	52.8	53.1	GBDP, 300mg, po, tid. Nitroglycerin is prescribed during angina attack.	Nitroglycerin is prescribed during angina attack.	Coronary heart disease angina	ukn	⑥ ⑦ ⑧ ⑨

Note: “T” = “experiment group”, “C” =“control group”, “m”= “months”, “w” =“weeks”, “d”=“days”, and “ukn”= “unknown”.

## Data Availability

The data supporting this systematic review and meta-analysis are from previously reported studies and datasets, which have been cited.
